# Year-Long Prevalence and Antibiotic Resistance Profiles of *Salmonella enterica* Serogroups Isolated from a Wisconsin Dairy Farm

**DOI:** 10.3390/pathogens13121031

**Published:** 2024-11-22

**Authors:** Courtney L. Deblois, Andrew D. J. Tu, Andrew J. Scheftgen, Garret Suen

**Affiliations:** 1 Department of Bacteriology, University of Wisconsin-Madison, Madison, WI 53706, USA; cdeblois@wisc.edu (C.L.D.); andrew.tu@wisc.edu (A.D.J.T.); scheftgen.a@gmail.com (A.J.S.); 2 Microbiology Doctoral Training Program, Department of Bacteriology, University of Wisconsin-Madison, Madison, WI 53706, USA; 3 Nutrition and Metabolism Graduate Program, Department of Nutritional Sciences, University of Wisconsin-Madison, Madison, WI 53706, USA

**Keywords:** dairy, *Salmonella*, transmission, prevalence, antibiotic, serogroup

## Abstract

*Salmonella enterica* infections can significantly impact the health and productivity of dairy cattle. Asymptomatic carriage of *Salmonella* can make it difficult to identify and monitor this pathogen across a herd. Therefore, a more focused *Salmonella* census on dairy farms is needed to better understand the dynamics of asymptomatic carriage. Here, we monitored the prevalence of *Salmonella enterica* on a dairy operation in Wisconsin, USA. Fecal samples were collected over 12 months from cattle and the farm environment, subjected to *Salmonella* isolation, serogrouped, and tested for antibiotic resistance. *Salmonella* was highly prevalent on this farm, with an average of 90% of the cattle being carriers. Total recovery of *Salmonella* from environmental samples ranged from 40 to 90%. Four serogroups were identified on the farm, with K being most common in cattle and C being most common in the environment. Antibiotic resistance was tested against eight antibiotics and was found to be highest for neomycin (44.5%) and sulfadimethoxine (86.3%). Our data show that serogroups associated with asymptomatic carriages are persistent and highly prevalent, with niche specificity to different locations. These results provide useful information for studying within-herd transmission of *Salmonella* and contributes to our understanding of transmission risks within the farm ecosystem.

## 1. Introduction

*Salmonella enterica* is a major enteric pathogen that causes significant disease in livestock. In dairy cattle, clinical signs of bovine salmonellosis can include diarrhea, dehydration, fever, abortion, and decreased milk production. Reduced milk yield, along with weight loss, mortality, and treatment expenses, can lead to substantial economic loss for dairy farms [1] that totals well over a billion dollars annually in the United States alone [2].

*Salmonella enterica* is a diverse species consisting of over 2000 serotypes with varying pathogenicity [3]. Previous literature has identified the most prevalent serovars associated with cattle as belonging to serogroups B, C, D, E, and K. Importantly, not all members of these serogroups are known to cause disease in cattle, and prior studies have shown that some serotypes are better adapted for asymptomatic colonization in the dairy environment. Historically, 40–60% of US dairy operations have tested positive for at least one serotype, and there are concerns that cattle asymptomatically colonized by *Salmonella* can shed these serotypes through feces into the farm environment [2]. However, without knowledge of these asymptomatic carriers, widespread environmental contamination can occur.

The spatial distribution of pens within a dairy farm plays a crucial role in the containment of pathogens like *Salmonella*. Pens located in close proximity to areas with higher levels of environmental contamination, such as sick and cull pens, are at an increased risk for harboring these bacteria. As such, identification of high-risk areas on the farm can inform targeted interventions to mitigate disease transmission and promote better overall health. Elucidating the significance of pen location on *Salmonella* prevalence on dairy farms is therefore essential for advancing our understanding of disease transmission and improving animal health. Taken together, it is clear that additional work is required to provide a better framework for producers to address the spread of *Salmonella* as it pertains to specific locations on the farm.

The ability of many *Salmonella* serovars to persist in the environment for extended periods is also of significant concern. Recent studies have shown that *Salmonella* can survive numerous farm processes including sand bedding recycling [4,5]. While sick and cull pens are known to harbor more *Salmonella*, relative to the rest of the farm, the temporal dynamics of serogroup distribution within these populations remain poorly understood. Furthermore, comparing serogroup distributions between the host and the environment is critical to understand persistence and transmission within the farm ecosystem. Although prior studies have investigated the prevalence and serovar distribution of *Salmonella* on healthy dairy operations, they are often cross-sectional or limited in duration. Long-term surveillance is likely required for capturing fluctuations in serogroup prevalence over time and represents a key gap in knowledge.

These concerns are further compounded by the increasing prevalence of antimicrobial resistance and the emergence of multi-drug resistance in *Salmonella* populations globally. Antimicrobial stewardship is contingent on the surveillance of antimicrobial resistance in the farm setting; thus, monitoring such resistance over time is the first step to combatting this issue. Here, we address the noted gaps in knowledge to understand the persistence of *Salmonella* on dairy farms with respect to time, location, and antibiotic resistance profiles. We accomplish this by tracking the prevalence of *Salmonella* within cattle and the farm environment over the course of a year on a dairy farm in Wisconsin, USA.

## 2. Materials and Methods

### 2.1. Study Design and Dairy Participation

This study was performed at the University of Wisconsin-Madison’s Emmons Blaine Dairy facility under the approved University of Wisconsin-Madison Institutional Animal Care and Use Committee Protocol #A006511. This facility is a mid-sized conventional Holstein dairy consisting of 430 milking cows, 100 dry cows, and over 50 calves under 10 weeks of age. This facility has five general lactating herd pens with free-stall housing bedded with recycled sand, a maternity and sick pen area with straw bed pack housing, and an outdoor calf area separate from the adult population. The calf area consists of individual hutch-style housing with straw bedding where calves are housed until weaning (6–8 weeks of age) whereupon they are moved to group-style hutches before being transported to a rearing facility.

### 2.2. Fecal Sample Collection

Fecal samples were collected from the facility once every two weeks over the course of a year. A total of 25 collection visits were conducted between March 2022 and February 2023. Appropriate personal protective equipment was worn at each collection, including site-specific boots, Tyvek suits (DuPont, Wilmington, DE, USA), and shoulder-length gloves (Nasco, Fort Atkinson, WI, USA) that were changed between collections. At each visit, eight pooled environmental samples were collected by sampling the four corners and the center of each pen and manually homogenizing the samples in a Whirl–Pak sampling bag (Nasco, Fort Atkinson, WI, USA). Additionally, 12 fresh fecal samples were collected via direct fecal grab from randomly selected healthy lactating members of the herd at each visit. All individual samples were transferred to 50 mL Falcon conicals (Corning, Glendale, AZ, USA) and placed on wet ice immediately after collection. Samples were transported to the lab, and culturing for *Salmonella* isolation began within 3 h of collection.

### 2.3. Salmonella Isolation and Identification

Isolation and identification of *Salmonella* isolates were performed as previously described [6,7]. Briefly, 1 g of feces was added to 15 mL Falcon conicals containing 10 mL of tetrathionate broth (Difco Laboratories, Sparks, MD, USA) activated with 200 μL of iodine (Weight (%), potassium iodide (20), and iodine (16)) (Remel (Thermo Fisher Scientific), Lenexa, KS, USA) and incubated overnight at 37 °C. Next, 100 μL of tetrathionate enrichment was added to 10 mL of Rappaport-Vassiliadis broth (Difco Laboratories, Sparks, MD, USA) and incubated overnight at 42 °C. Following enrichment, 10 μL of culture was streak-plated onto MacConkey (Difco Laboratories, Sparks, MD, USA) and XLT-4 agar (Difco Laboratories, Sparks, MD, USA) and incubated overnight at 37 °C. Colonies that produced hydrogen sulfide (as evidenced by black colony growth on XLT-4 agar) and found to be non-lactose fermenting (as evidenced by white colony growth on MacConkey agar) were further isolated and stored in glycerol and Luria-Bertani (LB) broth (Difco Laboratories, Sparks, MD, USA) at −80 °C until further use.

Isolates were confirmed as *Salmonella* positive via PCR of the *invA* gene, which is specific to *Salmonella*, as previously described [8] with the following modifications. Glycerol stocks were grown in 5 mL of LB broth overnight at 37 °C. Cell cultures were centrifuged at 3000× *g* for 15 min at 4 °C, decanted, and the pellets were resuspended in 2 mL of DNA extraction buffer (100 mM Tris-HCL, 10 mM EDTA, 0.15 M NaCl (pH 8.0)). Next, 1 mL of resuspension was added to a 1.5 mL tube and heated for 10 min at 60 °C. Samples were then centrifuged at maximum speed (14,000 rpm) for 10 min at 4 °C, decanted, and the pellets were resuspended in 50 μL of 1× Tris-EDTA buffer. PCR was performed by combining 1 μL of template from the previous step with 12.5 μL of GoTaqClear (Promega, Madison, WI, USA), 1 μL of forward primer (10 μM), 1 μL of reverse primer (10 μM), and 10.5 μL of nuclease free water. Thermocycler conditions were as follows: 95 °C for 3 min, 25 cycles of 95 °C for 30 s, 55 °C for 30 s, and 72 °C for 30 s, followed by a final 72 °C elongation for 5 min. PCR products were visualized on a 1% LE agarose gel (National Diagnostics, Atlanta, GA, USA) for 40 min at 100 V. Visual identification of bands with a size of 284 bp was used as confirmation of *Salmonella*. A PCR positive control of *Salmonella typhimurium* (ST4/74), as well as a PCR blank negative control (media with no culture added), was included. Due to financial constraints, confirmation of *Salmonella*-positive isolates were subjected to serogrouping using an antisera agglutination test for serogroups B (O:5), C (O:7,8), and K (O:18) according to manufacturer’s instructions (Cedarlane Labs, Burlington, NC, USA).

### 2.4. Antimicrobial Susceptibility Testing

All isolates confirmed as *Salmonella* were tested for antimicrobial susceptibility to the following eight antibiotics: ampicillin (10 μg), chloramphenicol (30 μg), enrofloxacin (5 μg), gentamicin (10 μg), neomycin (30 μg), oxytetracycline (30 μg), sulfonamide (300 μg), and trimethoprim/sulfamethoxazole (1.25/23.75 μg) (BD Diagnostic Systems, Franklin Lakes, NJ, USA). Testing for susceptibility to these antibiotics was performed by disc diffusion assays according to the methods described by the Clinical and Laboratory Standards Institute (CLSI) (CLSI, 2013, 2015). Sample 104 was lost prior to testing and thus was not subjected to antibiotic testing. Glycerol stocks were grown in 5 mL of LB broth overnight at 37 °C, 100 μL of overnight culture was spread-plated on tryptic soy agar with 5% sheep blood, stamped with discs, and incubated at 37 °C for 16–18 h prior to reading the results. Interpretive criteria for measuring zones of inhibition were based on CLSI standards. Isolates showing intermediate resistance were categorized as resistant.

### 2.5. Statistical Methods

All statistical tests were performed in R (R version 4.1.1 (10 August 2021)) using base R functions. Pairwise comparisons were performed using Fisher’s Exact test or a pairwise test of equal proportions when applicable, with a significance *p*-value level of <0.05.

## 3. Results

### 3.1. Salmonella Positivity Is Higher in Cows Than the Environment

**Figure 1:**
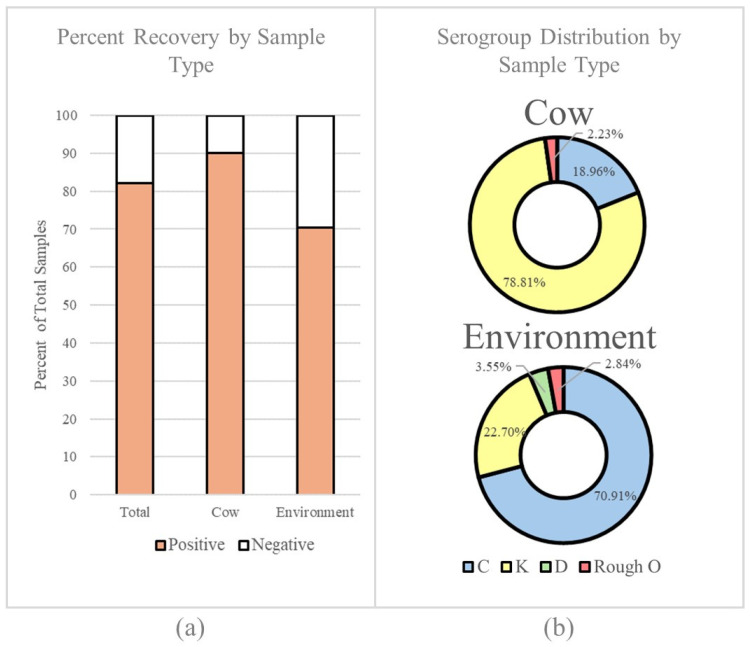
Summary of the *Salmonella* recovery rate and serogroup distribution throughout the study. (**a**) Percentage of samples from which *Salmonella* was recovered by sample type. (**b**) Pie charts outlining the serogroups identified on the farm by sample type.

A total of 499 fecal samples (*n* = 200 environmental samples and *n* = 299 direct fecal grabs) were collected over 25 sampling periods throughout the study. Of these, 82% were found to be positive for *Salmonella*, and 18% were negative. When enumerated by sample type, significantly more *Salmonella* was recovered from direct fecal grabs than from the environmental samples (*p*-value = 4.505 × 10^−8^ via Fisher’s Exact test), with a positivity rate of 90% (269 isolates) compared to 70.5% (141 isolates) (Figure 1).

**Figure 2:**
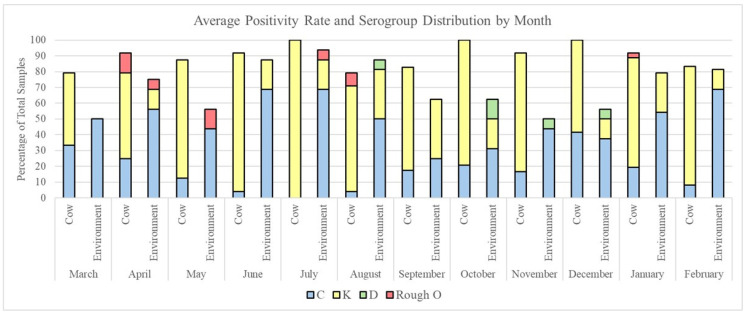
The percentage of samples from which *Salmonella* was recovered over time for both cow and environmental samples with serogroup distributions denoted by color.

The *Salmonella* positivity rate and serogroup distribution fluctuated over time for all sample types. Direct fecal grab samples maintained a high positivity rate throughout the year, averaging between 79.2 and 100% of the samples per month. Environmental samples showed the highest positivity in the months of June (87.5%), July (93.75%), and August (87.5%) and the lowest positivity in November (50.0%) (Figure 2). Comparisons between the positivity of direct fecal grabs and environmental samples by month showed significant differences in October (*p*-value = 0.005), November (*p*-value = 0.009), and December (*p*-value = 0.002) by pairwise tests of equal proportions.

### 3.2. Cows Are Dominated by Group K Serogroups Whereas the Environment Was Dominated by Group C Serogroups

Serogrouping of *Salmonella* isolates from direct fecal grab (*n* = 269) and environmental (*n* = 141) samples identified four serogroups: C, K, D, and rough O (Figure 1). Direct fecal grab samples were dominated by serogroup K at a percentage prevalence of 78.8%, which was significantly higher than that of the serogroup K isolates found in the environment (percent prevalence of 22.7%; *p*-value= 2.2 × 10^−16^ via Fisher’s Exact test). In contrast, environmental samples were dominated by group C isolates at a percent prevalence of 70.9%, while direct fecal grab samples had significantly less serogroup C isolates at 19.0% (*p*-value = 2.20 × 10^−16^ via Fisher’s Exact test). Rough O-presenting serotypes were identified in both direct fecal grab and environment samples in similar proportions (2.2% and 2.8% respectively). Serogroup D was only found in the environment at a prevalence of 3.6%.

### 3.3. Salmonella Serogroup Distribution in Cows vs. the Environment Varies over Time

We then sought to determine if serogroup positivity in our samples varied with respect to time. We found that, for our direct fecal grab samples, the majority of group K isolates were found in June and July, with positivity rates of 87.5% and 100%, respectively (Figure 2). This was significantly more than the number of group K isolates found in the months of March and April, which had the lowest overall positivity rates (45.8% and 54.2%, respectively). The opposite trend was observed for group C isolates, which exhibited the lowest positivity for the months of June (4.2%), July (0%), and August (4.2%) and the highest positivity for the months of March (33.3%) and December (41.7%).

In our environmental samples, the serogroup distribution was dominated by group C isolates, with the highest positivity in June (68.8%), July (68.8%), and February (68.8%) and the lowest positivity in September (25%), October (31.25%), and December (37.5%). In contrast, group K isolates were sporadic and low in the environment, as they were not found in the months of March, May, and November, but had the highest positivity in August (31.3%) and September (37.5%).

We then compared serogroup isolates between direct fecal grab and environmental samples according to month. We found group K isolates to be more prevalent in the direct grab fecal samples than in the environment in every month with the exceptions of August and September (all *p*-values > 0.05 via pairwise tests of equal proportions). Group C isolates were found to be more prevalent in the environment than in direct fecal grab samples in June, July, August, January, and February (all *p*-values < 0.05 via pairwise tests of equal proportions). The D and rough O-presenting serogroups were present in low abundance levels (total *n* = 5 and *n* = 10 respectively), with serogroup D only found in the environment in the months of August (*n* = 1), October (*n* = 2), November (*n* = 1), and December (*n* = 1). Rough O-presenting isolates were identified in both direct fecal grabs (April (*n* = 3), August (*n* = 2), and January (*n* = 1)) and the environment (April (*n* = 1), May (*n* = 2), and July (*n* = 1)). There were no significant differences in the prevalence of groups D and rough O within or between the direct fecal grab and environmental samples over time.

### 3.4. Salmonella in the Calf Environment Differs from That in the Adult Population Environment

**Figure 3:**
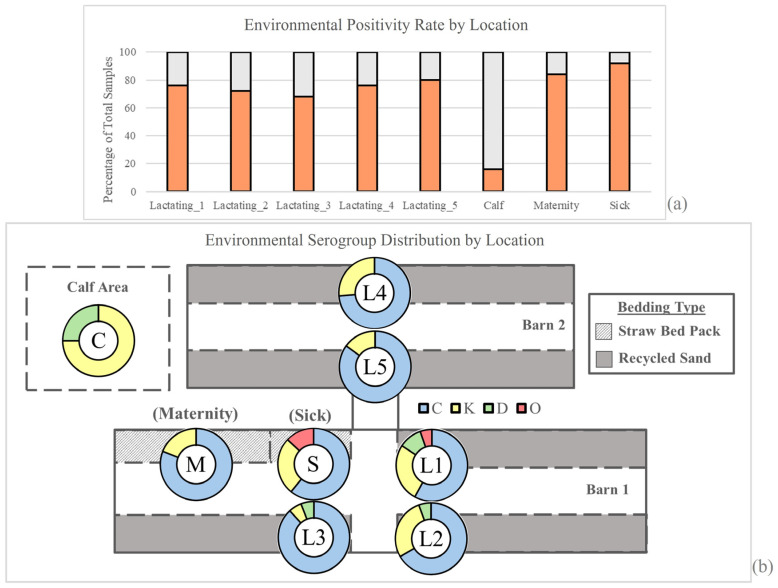
Summary of the farm layout, *Salmonella* recovery rate, and serogroup distribution from environmental samples. (**a**) Percentage of samples from which *Salmonella* was recovered by location. (**b**) Pie chart of serogroups obtained from environmental locations on the farm (see Supplementary Materials). Locations are designated as follows: C = calf area; M = maternity pen; S = sick pen, L1–L5 = lactating pens 1–5.

Of the 200 environmental samples collected over the course of our sampling year, 25 were from environmental locations including 5 lactating general herd pens, the sick pen, the maternity pen, and the calf area. Collection of bi-weekly samples resulted in a total of two sampling periods per month, with the exception of January 2023 (*n* = 3 sampling periods). Positivity rates and serogroup distributions were averaged across the year for each pen, resulting in the following positivity rates: Lactating pen (L) 1 = 76%, L2 = 72%, L3 = 68%, L4 = 76%, L5 = 80%, Calf = 16%, Maternity = 84%, and Sick = 92% (Figure 3). The calf area was found to have significantly less *Salmonella* relative to the environmental samples from the adult pens (all *p*-values < 0.001 via pairwise tests of equal proportions). All five general lactating herd pens had statistically similar positivity rates, averaging between 68 and 80% (all *p*-values > 0.05 via pairwise test of equal proportions). There were no significant differences in the percent recovery from the adult locations, including the sick and maternity pens.

### 3.5. Salmonella Positivity and Serogroup Distribution Vary by Location

Serogroup distributions did not differ significantly (all *p*-values > 0.05 via pairwise tests of equal proportions) across the environment of the adult population and consisted predominantly of group C isolates (Figure 3, L1 = 57.9%, L2 = 66.7%, L3 = 88.2%, L4 = 73.7%, L5 = 85.0%, M = 81.0%, and S = 60.9%) and group K isolates (L1 = 26.3%, L2 = 27.8%, L3 = 5.9%, L4 = 26.3%, L5 = 15.0%, M = 19.0%, and S = 26.1%). However, the calf area consisted of only serogroups K (75.0%) and D (25.0%) and had no group C isolates, similar to observations for the adult population (Figure 3). Additionally, the D and rough O-presenting groups were only found in pens that were close in proximity to one another.

### 3.6. Salmonella Was Resistant to Sulfonamides and Neomycin

**Figure 4:**
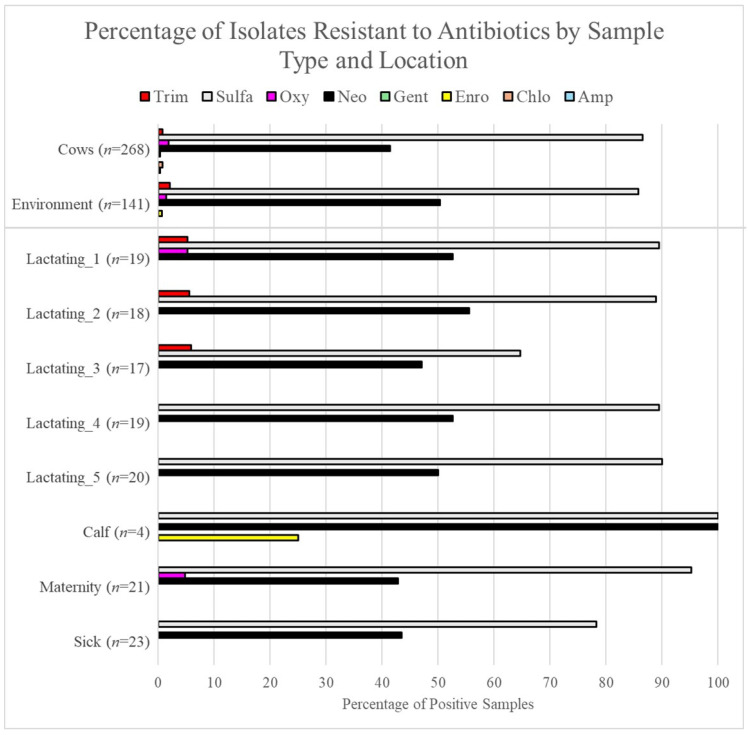
Antibiotic resistance of *Salmonella* isolates by sample type and location, as determined by disc diffusion assays. Isolates demonstrating intermediate growth against a given antibiotic were considered resistant. Antibiotics are designated as follows: Trim = trimethoprim sulfamethoxazole; Sulfa = sulfonamide; Oxy = oxytetracycline; Neo = neomycin; Gent = gentamicin; Enro = enrofloxacin; Chlo = chloramphenicol; Amp = ampicillin.

Antibiotic resistance was found to be highest against neomycin (neo) and sulfonamide (sulfa), averaging 44.5% and 86.3% of all isolates showing signs of resistance, respectively (Figure 4). A total of 1–2 isolates were sporadically resistant to other antibiotics (see Supplementary Materials), but there were no trends in antibiotic resistance between serogroup, time, location, or sample type.

**Figure 5:**
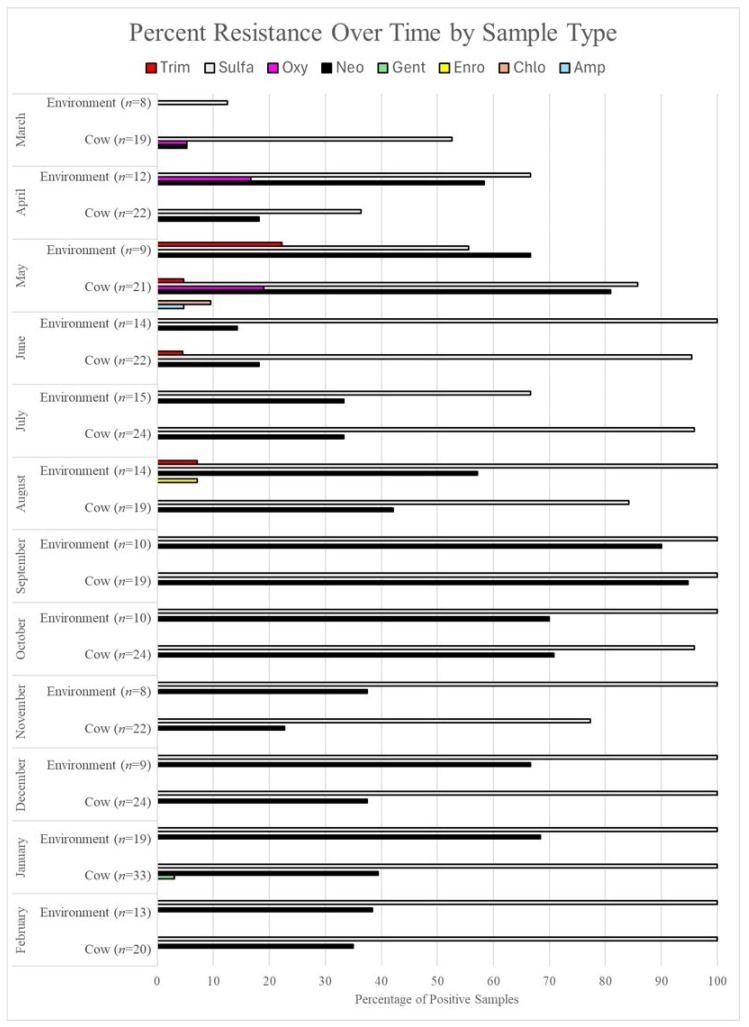
Antibiotic resistance of *Salmonella* isolates from cow and environmental samples over time, as determined by disc diffusion assays. Isolates demonstrating intermediate growth against a given antibiotic were considered resistant. Antibiotics are designated as follows: Trim = trimethoprim sulfamethoxazole; Sulfa = sulfonamide; Oxy = oxytetracycline; Neo = neomycin; Gent = gentamicin; Enro = enroflox-acin; Chlo = chloramphenicol; Amp = ampicillin.

Overall, antimicrobial resistance against sulfa was found to be the highest, with 86.6% of direct fecal grab isolates and 85.8% of environmental isolates exhibiting resistance. When enumerated over time, sulfa resistance was observed with the highest frequencies in September, December, January, and February, with 100% of the isolates showing resistance, whereas the lowest frequencies were found in April (36.4%) and March (52.6%) (Figure 5). For our environmental samples, resistance to sulfa was largely present year-round, with 100% resistance in June, August, September, October, November, December, January, and February, whereas the lowest percent resistance was found for March (21.5%), May (55.6%), April (66.7%), and July (66.7%). Comparisons of sulfa resistance between direct fecal grab and environmental samples revealed no significant differences in prevalence aside from July, where direct fecal grab isolates exhibited significantly more resistance than environmental isolates (cow = 95.8%, environment = 66.7%, *p*-value = 0.046 via pairwise tests of equal proportions). Within locations in the environment, sulfa resistance was most commonly found in the calf area and the maternity pen at percent resistances of 100% and 92%, respectively. The lowest percent sulfa resistance was found in lactating pen 3 (64.7%) and the sick pen (78.3%).

Neomycin resistance was also commonly observed across all samples, with 41.4% of direct fecal grab isolates and 50.4% of environmental isolates exhibiting resistance. Within direct fecal grab isolates, neo resistance fluctuated monthly from 5.3% in March to 94.7% in September. For environmental isolates, resistance also fluctuated across the year, from 0% in March to 14.3% in June and 90% in September. When comparing neo resistance between direct fecal grab and environmental isolates, there were no significant differences in prevalence aside from the month of April, where environmental isolates showed significantly more resistance than direct fecal grab isolates (environment = 58.3%, cow = 18.2%; *p*-value = 0.045 via pairwise tests of equal proportions). Within locations in the environment, neo resistance was highest in the calf area, with a percent resistance of 100%, and lowest in the maternity pens at 42.9%.

## 4. Discussion

*Salmonella* infections pose a significant threat to both animal and human health. The primary means of transmitting non-typhoidal *Salmonella* to humans is through food animals. Although the majority of research focuses on beef cattle operations, over three million culled dairy cows are slaughtered in the United States each year [9]. Previous research has shown that farm management plays a significant role in *Salmonella* transmission, although these practices vary largely between dairy and beef operations [10,11,12,13,14]. Therefore, it is critical to study *Salmonella* in the dairy farm setting to more fully understand pathogen transmission and recolonization cycles. Here, we sought to identify and monitor the prevalence of *Salmonella* serovars on a farm with no history of salmonellosis. Our study assessed the impact of location, time, and host on *Salmonella* prevalence, serogroup distribution, and antibiotic resistance.

Several studies have described the prevalence of fecal *Salmonella* shedding among dairy cattle to range between 2.3% and 22.5% depending on clinical presentation [6,7,15]. Here, we found a positivity rate of 82%, with 90% of direct fecal grabs and 70.5% of environmental samples containing *Salmonella*. The animals we sampled remained asymptomatic throughout the survey period, and there were no reported cases of salmonellosis during that time (Jessica Cederquist, personal communication). Although high *Salmonella* fecal shedding rates have been previously reported in beef cattle (70.9–73.7%) [16,17], to our knowledge, no dairy studies have reported a positivity rate of 90% among an asymptomatic general herd.

A number of factors can influence the positivity rate, including herd size, age, and manure management practices [14]. Previous studies have shown that a larger herd size is associated with increased positivity rates of *Salmonella* shedding [14,15,18]. We note that the farm in our study houses over 500 head, putting it at a higher risk for *Salmonella* transmission from animal to animal. We also found significant differences between the calf and adult populations. Previous studies have reported conflicting results with respect to which animal populations have the highest *Salmonella* positivity rates [15,18,19]. Calves are thought to have a higher *Salmonella* shedding rate due to their immature immune status. Together with their underdeveloped intestinal microflora, this puts them at a higher risk of infection. However, we found less *Salmonella* recovery in the calf population when compared to the adult population. We hypothesize that environmental/weather effects may be a likely reason, as calves are housed year-round in individual outdoor hutches. This is in contrast to adult cows, which are housed indoors in a free stall-style barn. We posit that the lack of animal-to-animal contact, as well as environmental exposure, is likely to reduce the survivability and transmissibility of pathogens, thereby resulting in lower positivity rates. However, we note that indoor housing and manure management practices have previously been documented as factors associated with higher positivity rates [14]. The farm in our study utilizes a sand reclamation system for bedding, and *Salmonella* has been shown to proliferate in sand bedding and survive the sand recycling process [4,5]. The continuous recycling of sand may play a significant role in the transmission cycle of *Salmonella* on our study farm; however, this remains an understudied topic.

In addition to overall *Salmonella* positivity rates, we also determined the serogroups of our isolates. The serogroups most commonly isolated from healthy lactating adults in this study belonged to groups C and K. Historically, group K serovars (such as *Salmonella* serovar Cerro) have presented subclinically and have not been associated with disease, whereas serovars belonging to group C (e.g., Kentucky, Montevideo, and Newport) have been known to cause salmonellosis [20,21]. The subclinical nature of the majority of the isolates collected throughout this study likely explains the high incidence rate we observed. Moreover, the lack of clinical presentation of disease from these serovars may allow undetected proliferation within the herd. We note that current management practices do not account for biosafety containment and screening of pathogens not known to cause disease. This has likely led to the rise of serovars K and C at a national level [2,21].

Analysis of the serotypes from our environmental samples also revealed a prevalence of group K and C isolates, which were dominated by serotype C. Serotype Cerro is the predominant serovar within serogroup K, and preliminary Kauffman–White testing of our isolates identified several belonging to Cerro. We note that the prevalence of Cerro has been on the rise, with one study of farms in Wisconsin, USA reporting an increase from 1% in 2006 to 37% in 2015 [2,21]. Importantly, this serovar has low pathogenicity due to mutations in several genes associated with virulence. These include a frameshift mutation in the *sopA* gene leading to a premature stop and the absence or partial absence of some *Salmonella* Pathogenicity Islands (SPIs) [22]. Despite this serovar’s reduced virulence and association with asymptomatic cattle, Cerro continues to grow in prevalence across dairy herds in the United States. This is likely due to a lack of effective control measures rather than increased host adaptation. Although advancements in identification and treatment strategies have led to decreases in pathogenic serovars, the prevalence of nonpathogenic serovars like Cerro continues to rise [21].

The increase in nonpathogenic serovars like Cerro coupled with a decrease in pathogenic serovars may be due to competitive exclusion. This concept posits that a given organism can competitively exclude the ability of another organism to thrive. This observation has been found for several *Salmonella* serovars in poultry, where non-pathogenic serovars can competitively exclude pathogenic serovars [23,24]. It is possible that this may also be occurring in dairy cattle, as evidenced by the cows in our study having a 90% recovery rate of *Salmonella* and no self-reported occurrences of salmonellosis on the farm during the study period. We note that our study found high amounts of potentially pathogenic serogroups, including those in group C, D, and rough O. The largest proportions of these serogroups were found in the environment. However, the cattle we sampled remained persistently colonized with group K throughout the study. We believe that the large proportion of group K *Salmonella* in cattle may be preventing colonization of potentially pathogenic serovars through competitive exclusion. As such, future research is needed to explore the possibility of competitive exclusion in a mammalian host.

Our data also suggests that transmission of serovars occurs between farm locations and that this is a function of proximity. For example, 9 of the 141 collected environmental isolates belonged to group D (*n* = 5) and rough O (*n* = 4). While the presence of these isolates is rare, they appeared in pens that were in close proximity to one another. For example, rough O was only found in the sick pen and lactating pen 1, which are physically separated by a small alley. The majority of group D isolates were found in pens (lactating pens 1, 2, and 3) separated by that same alley. This alley is subject to an automatic manure scraping system that carries waste to a large draining pit connecting these pens. We posit that this manure management system likely aids in the transmission of manure across pens, thereby transferring pathogens. However, little work has been conducted to consider the risk of *Salmonella* transmission with respect to manure management systems, and future studies in this area are warranted.

A key outcome of our year-long study is the ability to ascertain the impact of external factors on *Salmonella* prevalence. For example, we found that *Salmonella* prevalence in the environment is impacted by weather. The state of Wisconsin experiences weather conditions from below freezing in winter (November–March) to upwards of 40 °C in summer (June–August). Previous short-term studies have suggested that the *Salmonella* recovery rate is directly correlated with temperature [25,26]. Our results corroborate these findings with respect to environmental samples. This trend was not seen in fecal samples collected directly from the rectum of healthy lactating adult dairy cattle. This is perhaps due to the constant internal temperature maintained by these animals regardless of the ambient temperature. As such, these data suggest that environmental *Salmonella* recovery is reliant on temperature, while recovery directly from the host is not. This could be an important factor when considering management practices for the control of these pathogens.

Finally, the high prevalence of *Salmonella* on dairy farms is of significant concern due to the spread of antimicrobial resistance (AMR) linked to the increasing use of antimicrobials. Given that antimicrobial use is dynamic, our study provides an opportunity to gain insights into *Salmonella* AMR as it relates to antimicrobial use. Historically, *Salmonella* serogroups C and K, which constituted the majority of isolates observed in our study, have been reported to have relatively low antimicrobial resistance [2,21]. As such, we hypothesized that there would be no differences in antimicrobial resistance profiles over time and location due to their historically low resistance patterns. Consistent with our hypothesis, antibiotic resistance was found to be highest against neomycin and sulfadimethoxine. However, there was no correlation of antibiotic resistance with respect to sampling time, location, sample type, or serogroup, suggesting that antimicrobial resistance is stable across the two serogroups evaluated.

It is important to note that our finding of neomycin and sulfadimethoxine resistance does not correlate to the use of these antimicrobials on our farm (Jessica Cederquist, personal communication). Both neomycin (class aminoglycoside) and sulfadimethoxine (class sulfonamide) have seen significant declines in their use in the dairy industry. For example, there is a voluntary ban of aminoglycosides by several organizations including the American Veterinary Medical Association and the American Association of Bovine Practitioners. There are also imposed limits on the use of sulfonamides in the food animal sector by the U.S. Food and Drug Administration (FDA). Although previous studies have reported a significant decrease in aminoglycoside resistance since its voluntary ban [21], we found resistance in 44.5% of our isolates year-round. Similarly, sulfonamide use has decreased substantially, as it can only be used to treat bovine respiratory disease, hoof rot, and calf diphtheria. We posit that, although not used on our farm, serovars C and K may have developed resistance to both neomycin and sulfadimethoxine due to their widespread historical use across dairy farms. This likely resulted in these resistance genes becoming fixed within their genomes due to selective pressure against other antimicrobials within these classes. Given that ~20% of Wisconsin dairy farms continue to use sulfonamides for respiratory infections [27], it is plausible that these antimicrobially-resistant serovars are from outside sources, given that our farm is not closed.

## 5. Conclusions

In conclusion, our study presents a long-term investigation into how spatial and temporal variations impact *Salmonella* prevalence on a single farm. By sampling multiple environmental locations on the farm, as well as directly sampling animals over the course of a year, we were able to capture the dynamics of *Salmonella* over space and time that otherwise might be missed in single-timepoint or short-term studies. Our study thus provides a framework for understanding *Salmonella* carriage and spread across animals and their environment over time and further considers the impacts of external factors like weather and antimicrobial use on serovar dynamics. We believe this will be useful for developing strategies aimed at mitigating *Salmonella* infections in dairy herds in the future.

## Data Availability

The data that support the findings of this study are available from the corresponding author upon reasonable request.

## Supplementary Materials

The following supporting information can be downloaded at: https://www.mdpi.com/article/10.3390/pathogens13121031/s1, Table S1: Percent prevalence of serogroups by location; Table S2: Location AMR counts; Table S3: Cow and environment AMR counts; Table S4: Serogroup AMR counts.
