# Supplementary material for: Year-Long Prevalence and Antibiotic Resistance Profiles of Salmonella enterica Serogroups Isolated from a Wisconsin Dairy Farm
Source: Pathogens. 2024 Nov 22;13(12):1031. doi: 10.3390/pathogens13121031 (PMC11679656; doi:10.3390/pathogens13121031)
Supplement: Supplementary file 1 [file pathogens-13-01031-s001.zip › pathogens-3329194-supplementary.pdf]

Supplemental:

Supplemental Table S1: Percentage of *Salmonella* samples that were identified as each serogroup by location.

Percent Prevalence of Serogroups by Location				
	C	K	D	O
Lactating_1 (L1)	57.89%	26.32%	10.53%	5.26%
Lactating_2 (L2)	66.67%	27.78%	5.56%	0%
Lactating_3 (L3)	88.24%	5.88%	5.88%	0%
Lactating_4 (L4)	73.68%	26.32%	0%	0%
Lactating_5 (L5)	85.00%	15.00%	0%	0%
Calf (C)	0%	75.00%	25.00%	0%
Maternity (M)	80.95%	19.05%	0%	0%
Sick (S)	60.87%	26.09%	0%	13.04%

Supplemental Table S2: Raw counts of *Salmonella* isolates resistant to antibiotics by sample type and location, as determined by disc diffusion assays. Isolates demonstrating intermediate growth against a given antibiotic was considered resistant. Antibiotics are designated as follows: Trim = trimethoprim sulfamethoxazole; Sulfa = sulfonamide; Oxy = oxytetracycline; Neo = neomycin; Gent = gentamicin; Enro = enrofloxacin; Chlo = chloramphenicol; Amp = ampicillin.

Location AMR Counts									
	**Amp**	**Chlo**	**Enro**	**Gent**	**Neo**	**Oxy**	**Sulfa**	**Trim**	**Total**
L1	0	0	0	0	10	1	17	1	19
L2	0	0	0	0	10	0	16	1	18
L3	0	0	0	0	8	0	11	1	17
L4	0	0	0	0	10	0	17	0	19
L5	0	0	0	0	10	0	18	0	20
Calf	0	0	1	0	4	0	4	0	4
Maternity	0	0	0	0	9	1	20	0	21
Sick	0	0	0	0	10	0	18	0	23
Total	0	0	1	0	71	2	121	3	141

<u>Supplemental Table S3</u>: Raw counts of *Salmonella* isolates resistant to antibiotics by cow or environmental sample, as determined by disc diffusion assays. Isolates demonstrating intermediate growth against a given antibiotic was considered resistant. Antibiotics are designated as follows: Trim = trimethoprim sulfamethoxazole; Sulfa = sulfonamide; Oxy = oxytetracycline; Neo = neomycin; Gent = gentamicin; Enro = enrofloxacin; Chlo = chloramphenicol; Amp = ampicillin.

Cow AMR Counts									
	**Amp**	**Chlo**	**Enro**	**Gent**	**Neo**	**Oxy**	**Sulfa**	**Trim**	**Total**
March	0	0	0	0	1	1	10	0	18
April	0	0	0	0	4	0	8	0	22
May	1	2	0	0	17	4	18	1	21
June	0	0	0	0	4	0	21	1	22
July	0	0	0	0	8	0	23	0	24
August	0	0	0	0	8	0	16	0	19
September	0	0	0	0	18	0	19	0	19
October	0	0	0	0	17	0	23	0	24
November	0	0	0	0	5	0	17	0	22
December	0	0	0	0	9	0	24	0	24
January	0	0	0	1	13	0	33	0	33
February	0	0	0	0	7	0	20	0	20
Total	1	2	0	1	111	5	232	2	168
Environment AMR Counts									
	**Amp**	**Chlo**	**Enro**	**Gent**	**Neo**	**Oxy**	**Sulfa**	**Trim**	**Total**
March	0	0	0	0	0	0	1	0	8
April	0	0	0	0	7	2	8	0	12
May	0	0	0	0	6	0	5	2	9
June	0	0	0	0	2	0	14	0	14
July	0	0	0	0	5	0	10	0	15
August	0	0	1	0	8	0	14	1	14
September	0	0	0	0	9	0	10	0	10
October	0	0	0	0	7	0	10	0	10
November	0	0	0	0	3	0	8	0	8
December	0	0	0	0	6	0	9	0	9
January	0	0	0	0	13	0	19	0	19
February	0	0	0	0	5	0	13	0	13
Total	0	0	1	0	71	2	121	3	141

Supplemental Table S4: Raw counts of *Salmonella* isolates resistant to antibiotics by serogroup, as determined by disc diffusion assays. Isolates demonstrating intermediate growth against a given antibiotic was considered resistant. Antibiotics are designated as follows: Trim = trimethoprim sulfamethoxazole; Sulfa = sulfonamide; Oxy = oxytetracycline; Neo = neomycin; Gent = gentamicin; Enro = enrofloxacin; Chlo = chloramphenicol; Amp = ampicillin.

Serogroup AMR Counts									
	Amp	Chlo	Enro	Gent	Neo	Oxy	Sulfa	Trim	Total
C	0	0	1	0	68	2	127	2	150
K	1	2	0	1	106	5	218	3	244
D	0	0	0	0	3	0	5	0	5
Rough O	0	0	0	0	5	0	6	0	10
Total	1	2	1	1	182	7	356	5	409
